# Differences of Sex Development: A Study of 420 Patients from a Single Tertiary Pediatric Endocrinology Center

**DOI:** 10.3390/children12070954

**Published:** 2025-07-19

**Authors:** Silvia Ventresca, Laura Chioma, Rosario Ruta, Mafalda Mucciolo, Pasquale Parisi, Agnese Suppiej, Sandro Loche, Marco Cappa, Carla Bizzarri

**Affiliations:** 1 Department of Pediatrics, University Hospital Arcispedale Sant’Anna, University of Ferrara, 44124 Ferrara, Italy; 2 Pediatrics Unit, Department of Neuroscience, Mental Health and Sense Organs (NESMOS), Faculty of Medicine and Psychology, Sapienza University of Rome, 00185 Rome, Italy; 3 Unit of Pediatric Endocrinology, Bambino Gesù Children’s Hospital, IRCCS, Piazza S. Onofrio 4, 00165 Rome, Italy; 4 Translational Cytogenomics Research Unit, Bambino Gesù Children’s Hospital, IRCCS, 00165 Rome, Italy; 5 Research Unit for Innovative Therapies in Endocrinopathies, Bambino Gesù Children’s Hospital, IRCCS, 00165 Rome, Italy

**Keywords:** differences of sex development, gonadal dysgenesis, genetic diagnosis, next-generation sequencing

## Abstract

*Background*: Differences of sex development (DSD) are a group of congenital conditions characterized by atypical development of genital structures. The diagnosis is complex and involves clinical, hormonal, and genetic evaluations. *Objective*: To describe the clinical profile, diagnosis, and management of patients with DSD, with particular attention to genetic diagnosis. *Study design*: Retrospective study from a tertiary care pediatric hospital in Italy. *Methods*: 420 patients with DSD referred to the Endocrine Unit of Bambino Gesù Children’s Hospital in Rome, Italy, between 2016 and 2023 were included. *Results*: 75 patients had a 46,XY karyotype, 135 had a 46,XX karyotype, and 210 had chromosomal mosaicism. In our group of pediatric DSD patients, 21/420 patients were born from pregnancies induced with assisted reproduction techniques (ICSI/FIVET). Of these 21 patients, 5 had sex chromosome mosaicism. Using next-generation sequencing (NGS), we identified three new genetic variants: one in the *AR* gene, one in the *NR5A1* gene, and one in the *SRY* gene. The use of NGS significantly improved the diagnostic yield, and a definitive diagnosis was reached in 84.76% of the entire cohort. *Conclusions:* This study highlights the challenges in the management of patients with DSD from early recognition to treatment and follow-up. A multidisciplinary approach is essential for a comprehensive evaluation of these conditions and to understand the role and clinical significance of the genetic variants.

## 1. Introduction

Sex determination is a highly complex process involving many genetic pathways that results in translation of the sex chromosome complement (XX or XY) into the development of reproductive structures [1]. The term differences of sex development (DSD) refers to a heterogeneous group of congenital conditions affecting human sex determination and differentiation in which the development of chromosomal, gonadal, or anatomical sex is atypical [1], with a reported incidence in newborns of approximately 1/4500–1/5500 [2]. A comprehensive evaluation of atypical genitalia in a newborn is essential in order to understand the underlying cause and potential consequences [2].

DSD originate during embryonic and fetal development and can be the result of numerical or structural variations in sex chromosomes [1], variations in genes involved in gonadal and/or genital development [2], disorders in gonadal and/or adrenal steroidogenesis [3], maternal factors (endogenous or exogenous) [4], and endocrine disruptors affecting genital development [1,2,3,4,5]. Another possible category includes epigenetic changes affecting gene expression in the fetal period. In 2006, the Lawson Wilkins Pediatric Endocrine Society (LWPES) and the European Society for Paediatric Endocrinology (ESPE) published a consensus statement on the management of intersex disorders and the current classification of DSD based on the patient’s karyotype [3]. The classification includes three main diagnostic categories: sex chromosome DSD, 46,XY DSD, and 46,XX DSD. The category of sex chromosome DSD embraces not only 45, X/46,XY, 46XX/46XY mosaicisms and other sex chromosome mosaicisms/chimerisms, but also Turner’s syndrome (TS) and Klinefelter’s syndrome (KS). According to the current classification, patients with ovotesticular DSD—characterized by the presence of both ovarian tissue with follicles and testicular tissue with seminiferous tubules, either within the same gonad (defined as an ovotestis) or in opposite gonads (one ovary and one testis)—are categorized into different chromosomal DSD groups based on their karyotype: 46,XX, 46,XY, or mosaic/chimeric forms (e.g., 46,XX/46,XY) [3,4,5].

Our knowledge of DSD has greatly evolved in the past decade owing to cutting-edge research on mammalian sex development and the genetic mechanisms underlying DSD [1,2,3,4,5]. Molecular diagnosis is crucial for the appropriate management of a patient with DSD. In fact, the results of molecular investigation guide clinical, laboratory, and imaging investigations, and provide information on gonadal cancer risk, associated morbidity, and long-term outcomes. Molecular diagnosis is also important for genetic counseling and prenatal diagnosis [3,4,5].

In this study, we present the experience on the management and outcome of patients with DSD followed in our Pediatric Endocrinology Center from January 2016 to July 2023, and provide insight into the referral patterns, presentation, diagnoses, and associated features. We also share our experience regarding the usefulness of advanced genetic technologies in improving diagnostic accuracy.

## 2. Materials and Methods

### Patient Enrollment

The study involved 420 pediatric patients with DSD referred to the Outpatient Clinic for Pediatric Endocrinology of the Bambino Gesù Children’s Hospital in Rome between January 2016 and July 2023. All patients with DSD as defined by the current classification [3] participated in the study, including patients with KS and TS. Every single case was reviewed by a multidisciplinary team at our monthly meeting. Each case was also evaluated by our team of bioethicists, especially in situations involving uncertainty about sex assignment.

**Figure 1:**
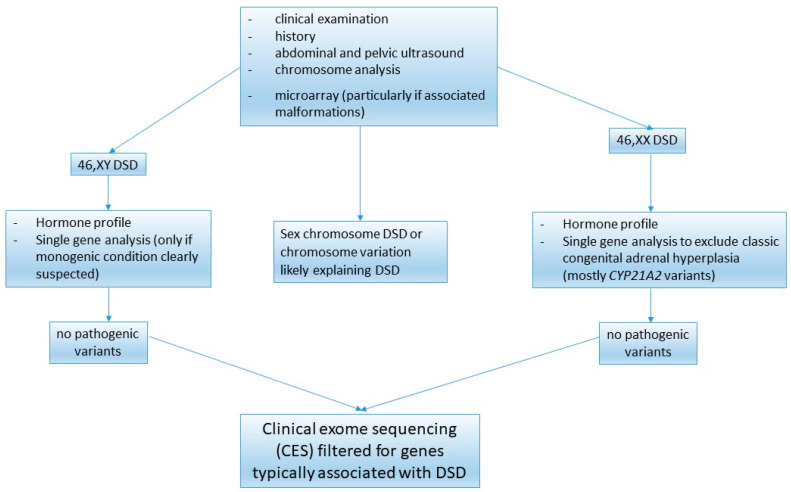
Clinical and genetic evaluation of newborns and children with DSD.

Peripheral blood karyotype analysis was performed to detect X and/or Y chromosomes and possible sex chromosomal mosaicisms. FISH analysis using specific probes was then performed to search for *SRY* sequences and Y rearrangements, when needed. No additional investigations were conducted in patients presenting with sex chromosome anomalies that explained the observed clinical phenotype. In all other cases, we carried out NGS using clinical exome sequencing (CES) filtered for genes typically associated with 46,XY DSD and 46,XX DSD (Figure 1). The list of genes is continuously updated according to scientific evidence. The broader CES approach allows for the sequence data of the entire exome for possible re-analysis, re-evaluation, and identification of new genotype–phenotype associations [6].

Measurement of 17-hydroxyprogesterone (17OH-P), dehydroepiandrosterone (DHEA), testosterone, follicle-stimulating hormone (FSH), luteinizing hormone (LH), ACTH, cortisol, renin, and anti-Mullerian hormone (AMH) was obtained in all patients at baseline. The present study includes both patients diagnosed before January 2020, whose gonadal steroid levels were measured using automated immunoassay methods, and more recent cases in which Liquid Chromatography–Tandem Mass Spectrometry (LC-MS/MS) was used to perform a full steroid profile.

All patients also underwent abdominal and pelvic ultrasound studies to assess the presence, location, and characteristics of the gonads, uterus, and vagina.

The Institutional Review Board approved the study protocol. Clinical data were collected, stored, and used following procedures in accordance with the Declaration of Helsinki (as revised in 2013) to guarantee the confidentiality and protection of data, with signed informed consents from the participating subjects/families.

## 3. Molecular Genetic Studies

High-resolution GTG chromosome banding was performed on peripheral blood samples collected in heparin vacutainers. Whole blood cultures were set up using RPMI 1640 media with fetal calf serum, antibiotics, and phytohemagglutinin M. GTG-banding involved trypsin and Giemsa dye immersion, followed by light microscopy analysis.

FISH was carried out on metaphase chromosome preparations using locus-specific BAC probes selected from the UCSC genome browser. The analysis was performed with an Eclipse 80i microscope (Nikon Europe B.V, Amstelveen, The Netherlands) and Genikon software (http://www.alphametrix.de).

For NGS, venous blood samples were collected, barcoded, and stored at −20 °C. DNA was extracted using the QIAsymphony DNA Mini Kit and sequenced using the NextSeq 550 and NovaSeq 6000 platforms. Sequencing By Synthesis (SBS) chemistry was used for DNA sequencing. Clinical exome sequencing (CES) filtered for genes typically associated with DSD was performed. The coding regions and exon–intron junctions of the following genes were analyzed: AMH (NM_000479), AMHR2 (NM_020547), AR (NM_000044.3), CBX2 (NM_005189.2), CYP11A1 (NM_000781), CYP17A1 (NM_000102), DHH (NM_021044.3), DMRT1 (NM_021951), FOXL2 (NM_023067), FST (NM_013409), GATA4 (NM_002052.4), HSD3B2 (NM_000198), HSD17B3 (NM_000197), MAP3K1 (NM_005921), NR0B1 (NM_000475.4), NR5A1 (NM_004959), SOX3 (005634), SOX9 (NM_000346), SRD5A2 (NM_000348), SRY (NM_003140), WNT4 (NM_030761), WT1 (NM_024426), WWOX (NM_016373.3), RSPO1 (001038633). DNA variants were annotated according to HGVS nomenclature and classified based on the standard of the American College of Medical Genetics and Genomics guidelines (ACMG) [6] as benign (class 1), likely benign (class 2), of uncertain significance (VUS, class 3), likely pathogenic (class 4), or pathogenic (class 5). Variant interpretation is based on scientific literature, as well as information from databases such as ClinVar, HGMD, LOVD, and gene- or disease-specific databases when available. Allele frequency is referenced from the population database gnomAD v2.1.1 and the laboratory’s internal database. Sanger sequencing was used to confirm identified variants and to conduct familial studies.

## 4. Results

The main clinical characteristics of our study population are summarized in Table 1. The mean age at first observation was 62.04 months (range 0–192 months), with significant differences related to the disease. All patients with classic congenital adrenal hyperplasia (CAH) or severe hypospadias were observed early after birth. All 10 patients with Mayer–Rokitansky–Kuster–Hauser (MRKH) syndrome were first observed at the end of puberty due to primary amenorrhea. The two patients with 46,XY DSD due to an *SRY* variant, one patient with 46,XX DSD due to a *WT1* variant, and one patient with 46,XX DSD related to a *WNT4* variant were first evaluated during the peripubertal period. The nine patients with Complete Androgen Insensitivity Syndrome (CAIS) first came to medical attention during infancy or early childhood. Twenty-one patients were born from pregnancies induced with assisted reproduction techniques (ICSI/FIVET). Thirteen patients also had other malformations, and twenty-seven underwent gonadectomy (Table 1).

Among the 420 patients, 75 had a 46,XY karyotype, 135 had a 46,XX karyotype, and 210 had chromosome mosaicisms. A total of 276 of these patients were assigned to the female sex (65.71%), and 144 (34.29%) were assigned to the male sex.

In the 46,XY DSD group (Table 2), 28 patients (37.33%) were assigned to the female sex. A total of 9 of these patients had CAIS, 3 had 17β-hydroxysteroid dehydrogenase type 3 (17βHSD3) deficiency, 8 had *SRD5A2* gene variants causing 5α-reductase deficiency, 2 had pathogenic variants in the *SRY* gene, and 6 had variants in the *NR5A1* gene causing a Steroidogenic Factor 1 (SF1) defect. In this group, 28 patients had severe hypospadias of unknown origin, and 2 patients had CAH due to 3 beta-hydroxysteroid dehydrogenase type 2 (3β-HSD2) deficiency.

Among the 46,XY DSD group, no pathogenic variants in known DSD genes were detected by NGS in 41 patients, representing 54.67% of the 46,XY DSD cohort.

Among the 46,XX DSD group (Table 3), 127 out of 135 patients were assigned to the female sex, and 8 were assigned to the male sex. In this group, 110 out of 135 patients had classic CAH, all presenting at birth with virilization of the external genitalia (Stage 4 Prader Score). Only one patient with 46,XX DSD due to CAH was assigned male at birth and shortly thereafter reassigned to female. Among non-CAH patients, 10 had MRKH syndrome, 5 had *SRY* translocation on the X chromosome, 1 patient carried a *WT1* variant, and 1 patient carried a *WNT4* variant. A definitive diagnosis was established in 117 out of 135 patients (86.67%) in the 46,XX DSD group, and in 7 out of 25 patients (28%) after exclusion of CAH. No definitive diagnosis was established in 17 patients from this group.

Among the sex chromosomal DSD group (Table 4), 89 out of 210 subjects were males (73 with KS syndrome and 16 with mixed gonadal dysgenesis, defined as a dysgenetic testis on one side and a streak gonad on the other), and 107 out of 210 patients were females with TS. A total of 42 girls with TS had X chromosome mosaicism.

Five of the 21 patients with DSD born from pregnancies induced with assisted reproduction techniques (ART) had sex chromosome mosaicism.

With the use of NGS, we were able to identify three new genetic variants:

- A novel hemizygous variant of the androgen receptor (*AR*) gene was identified in a patient with 46,XY DSD who was assigned female at birth and referred in infancy for bilateral inguinal hernias containing gonads: NM_000044.3: c.2424G>T, p.Met808Ile. This missense variant segregated from the mother and has not yet been reported in the scientific literature; it can be considered a Variant of Uncertain Significance (VUS), meaning a sequence variant with unknown functional and clinical impact, but family history revealed one family member (mother’s sister) clinically affected by CAIS in the absence of a definitive genetic diagnosis.
- A novel variant of the *NR5A1* gene was found in a boy with 46,XY DSD who presented with penoscrotal transposition, hypospadias, and cryptorchidism. This boy carried a de novo heterozygous variant: NM_004959: c.[1096C>T];[=] (p.[(Q366*)];[(=)]). At the protein level, the variant resulted in p.Gln366Ter, leading to the formation of a premature stop codon. This variant has not been reported in the scientific literature; however, its potential impact on protein function suggests a possible association with the clinical indication for testing.
- The third novel variant was found in a girl with a 46,XY pure gonadal dysgenesis. Analysis of the *SRY* gene revealed a de novo heterozygous variant: NM_003140.3 (SRY): c.[359A>G];[0] p.[(His120Arg)];[0] at the level of the HMG-box region. There is no evidence in the literature or major databases (e.g., ClinVar) confirming this variant as pathogenic. It has not been reported as benign either. Therefore, based on current knowledge, it remains a VUS—a missense variant with uncertain clinical and functional impact. From a clinical perspective, this result was compatible with the clinical picture of pure gonadal dysgenesis.

## 5. Discussion

Our study investigates the spectrum and clinical characteristics of 420 patients diagnosed with DSD at an Italian pediatric tertiary care center. It focuses on incidence rates, clinical presentation, and genetic diagnosis, representing the largest Italian study conducted on young patients with DSD to date.

In our patient series, the number of affected females (276) was higher than that of males (144).

In the 46,XY DSD group, the most frequent condition was severe hypospadias, followed by patients with no genetic diagnosis and different disorders of androgen synthesis or action (CAIS/PAIS, 5α-reductase deficiency, *SF1* deficiency, 17 beta HSD3 deficiency, *SRY* variant, and CAH due to HSD3β2 deficiency). In a recent study from Turkey, 5α-reductase deficiency was identified as the most common cause of 46,XY DSD, followed by CAIS and PAIS [7]. Ethnic differences may account for these conflicting results.

As previously observed [8,9,10], we found that 46,XX DSDs are more common than 46,XY DSDs due to the high prevalence of CAH, with the sex chromosomal DSD group representing the largest category [3,11,12,13,14,15,16]. Similar to other case series [17,18,19], in our study, nearly all cases of the 46,XX group were caused by CAH due to 21-hydroxylase deficiency. Despite its high prevalence, some authors have recently questioned the inclusion of CAH among the DSDs, given that the gonads are unaffected in this condition [20,21]. The incidence of CAH, particularly the classic form, is approximately 1 in 10,000 to 1 in 15,000 live births, whereas KS has an estimated incidence of 1 in 600 to 1000 live male births [3,4]. However, in our case series, the prevalence of CAH was higher than that of KS. It is well known that KS may remain undiagnosed until adulthood, which could explain the findings in our cohort that necessarily has a selection bias (children referred to a tertiary center for pediatric endocrinology).

The clinical presentation of DSD varied widely in our study, with symptoms ranging from severely atypical genitalia and hypospadias to amenorrhea and delayed puberty. The age at presentation also varied, spanning from the neonatal period to late adolescence. The median age at presentation across all cases was 5.17 years, with younger ages observed in the 46,XX DSD group with CAH compared to other groups, consistent with previous reports in the literature [22,23]. The age at diagnosis of 46,XY DSD depended primarily on the underlying etiology, and delayed diagnosis was more common in this group. However, the median age at CAIS diagnosis in our cohort was younger than that reported in the literature [24,25,26].

The reasons for referral aligned with previous reports [27,28]. In fact, 84.4% of patients with DSD 46,XX and 56% of patients with 46,XY DSD were referred for atypical genitalia, while patients with sex chromosome DSD were rarely referred for this reason. Factors related to ethnicity and/or social or cultural environment may also play a role in delaying the diagnostic process [11,12,13]. The perception of gender and expectations, as well as the phenotypic appearance of affected individuals, may be overlooked or underestimated, leading to significant diagnostic delays and confusion during medical assessment [11,12,13].

Decisions regarding gender assignment vary depending on the specific condition. In cases of 5α-reductase (5αRD2) deficiency and possibly 17β-hydroxysteroid dehydrogenase (17βHSD3) deficiency diagnosed in infancy, the predominance of male gender identity among patients and the potential for fertility (documented in 5αRD2, but uncertain in 17βHSD3) should be carefully considered when determining gender assignment [3]. Currently, reassignment to male gender is strongly recommended for newborns and infants with 5α-reductase deficiency who were assigned male at birth, while assignment to female gender is recommended for virilized newborn girls with 46,XX DSD due to CAH [3]. Since our hospital does not have an obstetrics unit, newborns with DSD are referred to our center after birth, with their legal sex typically already assigned at the birth hospital. Notably, only one patient with 46,XX DSD due to CAH was assigned male at birth and shortly thereafter reassigned to female on the basis of the advice of our multidisciplinary team. Three 46,XY patients with 5α-reductase deficiency were assigned male at birth and underwent early surgical correction of the genitalia accordingly, while two out of five children who were assigned female at birth were later reassigned to male. All patients with CAIS were assigned female at birth, whereas all patients with PAIS were assigned male.

Seven patients in the group with mixed gonadal dysgenesis underwent gonadectomy for the presence of a dysgenetic testis on one side and a streak gonad on the other. Gonadectomy is still a debated issue in the management of individuals with DSD, especially in those with mixed gonadal dysgenesis [29,30,31]. Dysgenetic testicles and streak gonads associated with DSD harbor a significantly higher risk of neoplasia. Specific tumors such as gonadoblastoma, dysgerminoma, and other germ cell tumors are frequently observed in these conditions [29]. The decision to perform gonadectomy is largely driven by the malignancy risk, which stems from abnormal germ cell maturation and predisposing factors such as the gonadal intraepithelial neoplasia spectrum [29,30,31]. Prophylactic removal of the gonads has traditionally been recommended promptly after the diagnosis of CAIS is confirmed [32]. Since the first description of the condition by Morris [33], the estimated risk of gonadal malignancy has been reported to vary widely, from as high as 22% to as low as 0.8% [34]. Therefore, current guidelines [3,4,35] recommend delaying the procedure until after spontaneous puberty, due to the benefits of endogenous hormone production during pubertal development. A recent review proposed an algorithm for managing CAIS beginning in adolescence, emphasizing a conservative approach for patients who choose not to undergo gonadectomy [36]. Up to 15% of CAIS patients retain their gonads after puberty, but the risk of germ cell tumors increases with advancing age [34].

The timing of gonadectomy is also a critical aspect of DSD management. During puberty, elevated gonadotropin levels, particularly FSH, are known to have tumor-promoting effects and further increase the malignancy risk [34,35,36,37]. Consequently, early prophylactic gonadectomy is generally recommended in cases where the risk of gonadoblastoma—a precursor to dysgerminoma and other high-grade germ cell tumors—is significant. In contrast, for individuals with functional gonads and low immediate malignancy risk, careful surveillance with regular imaging and tumor marker monitoring may be considered [34,35,36,37].

Within our cohort, a definitive diagnosis was obtained in only 36% of patients, following the exclusion of those with CAH and sex chromosomal DSD, the latter of which can be diagnosed based solely on karyotype. A novel variant in the *AR* gene was found in a patient with CAIS. A novel de novo heterozygous variant of the *NR5A1* gene was identified in a boy with 46,XY DSD presenting with penoscrotal transposition, hypospadias, and cryptorchidism. A novel *SRY* variant was found in a girl with a 46,XY karyotype and pure gonadal dysgenesis (Swyer syndrome). The three novel variants were classified as variants of uncertain significance (VUS), potentially limiting their clinical utility. However, the variant in the *AR* gene was inherited from the mother, whose sister was clinically affected by CAIS, even though a definitive genetic diagnosis was not established, supporting the potential pathogenicity of the variant. The variant identified in the NR5A1 gene was de novo and introduced a premature stop codon; both factors contributed to supporting its classification as likely pathogenic in accordance with current guidelines. The novel variant in the *SRY* gene was de novo, located in the critical HMG-box region, and associated with a compatible clinical phenotype; all these factors increase the likelihood that it is pathogenic. These findings highlight the importance of NGS in the diagnosis of complex DSD cases [38].

Our findings are based on a substantial and heterogeneous DSD cohort, which strengthens the generalizability and relevance of our clinical insights. The results are, in general, consistent with the existing literature, but there are some limitations. In particular, NGS did not yield diagnostic variants in 13.33% of cases with 46,XX DSD and 54.67% of cases with 46,XY DSD. This highlights the need for further genetic investigation, such as clinical exome sequencing (CES), to identify novel candidate genes involved in the development of the reproductive system [39]. Future research focusing on these genes and their functions is likely to improve diagnostic accuracy and clinical management strategies for individuals with DSD.

In our case series, 21 patients were born from pregnancies achieved through ART, and 5 of them had sex chromosome abnormalities. A slightly increased risk of chromosomal and genetic abnormalities, as well as malformations, has been reported in children born from ART pregnancies, mainly influenced by factors related to infertility and likely not by the procedure itself [40,41]. The age of the parents and genetic issues related to infertility largely explain this increase [42,43,44]. Approximately 3% of couples undergoing Intracytoplasmic Sperm Injection (ICSI) present chromosomal abnormalities (2.7% of men and 13% of women), underscoring the value of karyotyping prior to treatment [40].

In conclusion, we have presented our experience on a large cohort of patients with DSD, confirming The broad spectrum of clinical presentations and the significant genetic variability. The journey from initial diagnosis to long-term care remains intricate and requires a multidisciplinary and patient-centered strategy tailored to individual needs [45]. Clinical exome sequencing (CES), filtered for genes typically associated with DSD, has proven to be a robust and efficient diagnostic tool, underscoring the important role of advanced genetic technologies in enhancing diagnostic precision and guiding personalized care for individuals with DSD.

## Figures and Tables

**Table 1:** Main characteristics of the study population.

		Total(n)	Male Social Sex(n)	Mean Age at First Endocrinology Visit (Months)	Associated Malformations n (%)	Gonadectomy n (%)
**46,XY DSD**	NR5A1 variant (SF1 deficiency)	6	1	15.17	0 (0)	4 (80.00)
	Complete androgen insensitivity (CAIS)	9	0	17	0 (0)	6 (60.00)
	Partial androgen insensitivity (PAIS)	4	4	9.25	1 (25.00)	1 (25.00)
	Congenital adrenal hyperplasia (CAH)	2	2	0	0	0
	17β-HSD3 deficiency	3	1	54	0	2 (66.67)
	SRD5A2 variant (5α-reductase deficiency)	8	3	8.1	0	2 (28.57)
	SRY variant	2	0	192	0	1 (50.00)
	Severe hypospadias of unknown origin	28	28	0	0	0
	46,XY DSD of unknown origin	13	8	77	1 (7.69)	2 (15.38)
**46,XX DSD**	Mayer–Rokitansky–Kuster–Hauser (MRKH) syndrome	10	0	152.63	4 (40.00)	0
	Congenital adrenal hyperplasia (CAH)	110	0	0	0	0
	SRY translocation on X chromosome	5	5	0	0	0
	WT1 variant	1	0	120	0	1 (100)
	WNT4 variant	1	0	192	0	0
	46,XX DSD of unknown origin	8	3	115.7	2 (25.00)	1 (12.50)
**Sex chromosomal DSD**	Turner syndrome	65	0	70	1 (1.54)	0
	Klinefelter syndrome	73	73	51	1 (1.37)	0
	Turner syndrome mosaicism	42	0	82	0	0
	Mixed gonadal dysgenesis	30	16	24	3 (10.00)	7 (23.33)

**Table 2:** 46,XY DSD group.

	Tot	46,XY DSD of Unknown Origin	SF1 Defect	CAIS	PAIS	CAH (3β-HSD2 Deficiency)	17β-HSD3 Deficiency	5α-Reductase Deficiency	SRY Variant	Severe Hypospadias of Unknown Origin
**n (%)**	75	13 (17.33)	6 (8.00)	9 (12.00)	4 (5.00)	2 (2.67)	3 (4.00)	8 (10.67)	2 (2.67)	28 (37.33)
**Genetic variant**		-	*NR5A1*	*AR*	*AR*	*HSD3β2*	*17β−HSD3*	*SRD5A2*	*SRY*	*-*
**No molecular diagnosis (%)**	41 (54.67)	13 (100)	0 (0)	0 (0)	0 (0)	-	0 (0)	0 (0)	0 (0)	28 (100)
**Neonatal genital phenotype**		3 female 8 atypical genitalia 2 male	1 atypical genitalia 4 females 1 male	9 female	4 atypical genitalia	2 atypical genitalia	2 female 1 male	4 female 3 male 1 atypical genitalia	2 female	26 atypical genitalia 2 male
**Male social sex**	47	8 (61.54)	1 (16.67)	0 (0)	4 (100)	2 (100)	1 (33.33)	3 (37.5)	0 (0)	28 (100)
**Mean age at first endocrinology visit**		77 months	15.17 months	17 months	9.25 months	0 months	54 months	8.1 months	192 months	0 months
**Mean age at** **molecular diagnosis**		-	62.00 months	38.5 months	16 months	2.4 months	57.33 months	11.1 months	192 months	NA
**Spontaneous** **puberty**		3 yes 3 no 7 NA	3 no 3 NA	1 yes 2 NA 6 no	3 no 1 NA	2 NA	1 NA 2 no	3 no 5 NA	1 yes 1 no	26 NA 2 yes
**Genital phenotype at puberty**		6 female 7 NA	3 NA 3 female	7 female 2 NA	3 male 1 NA	2 NA	2 female 1 NA	3 female 5 NA	2 female	26 NA 2 male
**Gonads**		6 testicles 2 ovary/ testicle 4 streak gonads 1 ovaries	4 testicles 2 streak gonads	9 testicles	4 testicles	2 testicles	3 testicles	8 testicles	2 streak gonads	28 testicles

**Table 3:** 46,XX DSD group.

	**Tot**	**46,XX DSD of Unknown Origin**	**MRKH Syndrome**	**CAH**	**SRY Translocation on X Chromosome**	**WT1 Variant**	**WNT4 Variant**
**n (%)**	135	8 (5.93)	10 (7.41)	110 (81.48)	5 (3.70)	1 (0.74)	1 (0.74)
**Genetic variant**		-	*-*	*CYP21A2*	*-*	*WT1*	*WNT4*
**No molecular diagnosis (%)**	18 (13.33)	8 (100)	10 (100)	0 (0)	0 (0)	0 (0)	0 (0)
**Neonatal genital phenotype**		3 female 1 male 4 atypical genitalia	10 female	110 atypical genitalia	5 male	1 female	1 female
**Male social sex (%)**	8 (5.93)	3 (37.50)	0 (0)	0 (0)	5 (100)	0 (0)	0 (0)
**Mean age at first endocrinology visit**		115.70 months	152.63 months	0 months	0 months	120 months	192 months
**Spontaneous puberty**		3 yes 4 no 1 NA	10 no	43 yes 67 NA	3 yes 2 NA	1 no	1 yes
**Genital phenotype at puberty**		3 female 4 male 1 NA	10 female	43 female 67 NA	3 male 2 NA	1 female	1 female
**Gonads**		4 testicles 1 ovaries 1 ovary/testicle 2 streak gonads	10 ovaries	110 ovaries	5 testicles	1 streak gonads	1 ovaries

**Table 4:** Sex chromosome DSD group.

	Tot	Turner Syndrome	Turner Syndrome Mosaicism	Klinefelter Syndrome	Mixed Gonadal Dysgenesis
**n (%)**	210	65 (30.95)	42 (20.00)	73 (34.76)	30 (14.29)
**Neonatal genital phenotype**		65 female	42 female	73 male	11 atypical genitalia 12 female 7 male
**Male social sex** **(%)**	89 (42.38)	0 (0)	0 (0)	73 (100)	16 (53.33)
**Mean age at first endocrinology visit**		70 months	82 months	51 months	24 months
**Spontaneous puberty**		2 yes 29 NA 34 no	5 no 16 NA 21 yes	15 NA 40 yes 18 no	13 yes 12 NA 5 no
**Genital phenotype at puberty**		65 female	16 NA 26 female	73 male	13 female 12 NA 5 male
**Gonads**		65 ovaries	42 ovaries	73 testicles	9 testicles 14 ovaries 7 streak gonads

## Data Availability

The original contributions presented in this study are included in the article. Further inquiries can be directed to the corresponding author.
